# Validating the use of Medicare Australia billing data to examine trends in skin cancer

**DOI:** 10.12688/f1000research.7161.1

**Published:** 2015-11-24

**Authors:** Eshini Perera, Neiraja Gnaneswaran, Marlon Perera, Rodney Sinclair

**Affiliations:** 1 Cancer Council Victoria, Melbourne, Australia; 2 Faculty of Medicine, Dentistry and Health Science, University of Melbourne, Parkville, Australia; 3 Plastic Surgery, Royal Brisbane Hospital, Brisbane, Australia; 4 School of Medicine, University of Queensland, Brisbane, Australia; 5 Sinclair Dermatology, Melbourne, Australia

**Keywords:** Nonmelanoma skin cancer, Dermatology incidence, epidemiology, melanoma, medicare

## Abstract

**Background:  **Epidemiological data surrounding non-melanomatous skin cancer (NMSC) is highly variable, in part due to the lack of government cancer registries. Several studies employ the use of Medical Australia (MA) rebate data in assessing such trends, the validity of which has not been studied in the past. Conversely, melanoma skin cancer is a notifiable disease, and thus, MA and cancer registry data is readily available. The aim of the current study is to assess the use of MA for epidemiological measures for skin cancers, by using melanoma as a disease sample.

**Methods:**  Following ethics approval, data from MA and Victorian Cancer Registry (VCR) from 2004-2008 were extracted. Incidence of MA and VCR unique melanoma cases were compared and stratified by age and local government area (LGA). Regression and a paired-samples t-test were performed.

**Results:** During the study period; 15,150 and 13,886 unique melanoma patients were identified through VCR and MA data sources respectively. An outlier in the >80­ year age group was noted between MA and VCR data. When stratified by age, significant correlation between MA and VCR was observed for all patients (gradient 0.91, *R²= 0.936*) and following exclusion of >80 patients (gradient 0.96, *R²= 0.995*). When stratified by LGA, a high degree of observation was observed for all patients (gradient 0.94, *R²= 0.977*) and following exclusion of >80 patients (gradient 0.996, *R²= 0.975*).

**Conclusion: **Despite the inclusion of outlier data groups, acceptable correlation between MA and VCR melanoma data was observed, suggesting that MA may be suitable for assessing epidemiological trends. Such principals may be used to validate the use of MA data for similar calculations assessing NMSC trends.

## Introduction

Non-melanoma skin cancers (NMSC) are the most commonly diagnosed cancer in Australia. In Australia, excluding Tasmania, no government cancer registries record information regarding NMSC ^1^. Incidence and prevalence data surrounding NMSC is difficult to collect and results are highly variable ^2– 4^. Previously in Australia, NMSC epidemiological data has been obtained through large-scale prospective surveys and clinical examinations ^1, 3– 21^. Recently, studies examining the costs and rates of NMSC services have employed the use of the Medicare Australia (MA) database of item numbers billed ^2, 22^. Furthermore, studies examining other cancer trends also examine MA data in a similar way ^23– 25^. The MA data source has the potential to provide a very large amount of information concerning skin cancer trends in Australia. No previous study has validated the use of MA data for epidemiological cancer calculations. Thus there is a need to demonstrate that the level of ascertainment of cases captured by MA is sufficient to allow for meaningful research of skin cancer using this dataset.

In Australia, melanoma is a notifiable cancer, with state-based cancer registry data ^26^. Further, MA billing data exists for the management of malignant melanoma. The availability of such data provides the possibility of validating the use of MA data for epidemiological measures of melanoma. The aim of this study was to assess the accuracy of MA’s Medical Benefits Schedule (MBS) rebate data for incidence and patterns of melanoma skin cancer in Australia. The methodology used to validate the use of MA to calculate incidence may represent the possibility of using MA data to examine other types of skin cancer trends in Australia.

## Methodology

The Human Resources and Ethics Committee (HREC) granted approval to the Cancer Council of Victoria (CCV) on 1/10/2008 by the Department of Health and Ageing and was given the reference number 2008/CO004599. Ethics approval was for the use of MA data to be examined by the CCV for epidemiological purposes. Access to the Victorian Cancer Registry (VCR) dataset was released under the ‘Memorandum of Understanding.’ The data release was approved by the Director of the VCR on 9/7/2013. HREC approval was not required because only aggregated de-identified data was requested.

### Patient selection and data collection

The following item numbers pertaining to excision of malignant melanoma and *in situ* melanoma were extracted for use from the MA dataset: 31300, 31305, 31310, 31315, 31320, 31325, 31330, 31335. The data available for analysis included age group, gender, local government area (LGA) in which the patient was located, grouped location of tumour removal and tumour sizing (grouped as < 10mm, = 10mm or > 20mm). The MA criteria for these item numbers includes excision of both malignant and *in situ* melanoma (labelled as ‘Hutchinson’s melanotic freckle’ in the MA definition) ^27, 28^. Melanomas that were both malignant and *in situ* were extracted from the VCR database for the period of 2004 to 2008. Melanoma data registered with the VCR over the years 2004 to 2008 was also extracted for use in the study. Data available included age of patient, gender, LGA, *in situ* or malignant tumour and thickness level of tumour. Lastly, population data, from the Australian Bureau of Statistics (ABS) was used. Population data for the years studied was extracted for each LGA in Victoria.

The number of unique patients, that is, the number of different individuals requiring one or more melanoma treatment(s) was determined for the MA and VCR dataset. The number of unique patients in each LGA was then examined in the following components: VCR and MA dataset; VCR and MA dataset stratified by year; VCR and MA dataset stratified by gender; and VCR and MA dataset stratified by age group.

### Data analysis

Ordinary least squares’ (OLS) regression was used to analyse both datasets in each of the stratifications. In all of the OLS regressions performed, the *y*-axis intercept ( *b*) was never significantly different from zero. Therefore the simplest formula *y = mx* was used to describe the relationship between VCR and MA data across LGAs. Using the OLS regression method, the standard uncertainty in the gradient ( *u(m)*) and the Pearson’s correlation coefficient squared *(R ^2^)* values were obtained.

Incidence rates were calculated for each LGA with ABS data. The mean difference in incidence rates between the following ‘pairs’ were calculated across LGAs: VCR vs. MA incidence for each of the study years between 2004 and 2008; VCR vs. MA total population; VCR vs. MA incidence for males and females; and VCR vs MA incidence for each of the stratified age group: 0–19, 20–29, 30–39, 40–49, 50–59, 60–69, 70–79 and >80.

The paired sample t-test was used to test whether the mean difference in incidence between each of these pairs was greater than zero. The mean difference for each VCR-MA pair calculated and the 95% confidence interval for each pair was produced. A 2-tail significance test was performed to determine if the mean difference was statistically significant. All statistical analysis was carried out by IBM SPSS v.20.

## Results

A total of 15,150 unique patients with malignant melanoma and *in situ* melanomas were registered with the VCR between 2004 and 2008. During the same time period, MA was billed for 13,886 patients requiring melanoma treatment services. During the study period, the number of unique cases registered remained relatively stable for both datasets.

**Figure 1.**
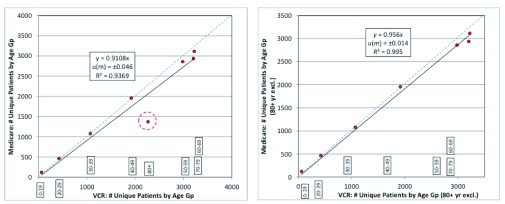
OLS regression of MA data against VCR data – stratified by age. ( **a**) including all patients ( **b**) excluding patients >80 years old.

During preliminary analysis it was noted that the number of melanoma cases did not correlate closely between both datasets in the 80+ year age group. Table 1 summarises the number of unique cases registered with VCR and MA resolved by gender and including and excluding patients in the 80+ year age group. Comparisons of both datasets stratified by age group revealed that the 80+ year age group was a clear visible outlier ( Figure 1). The standard of uncertainty was significant at ± 5% ( *u(m)* ±0.046). Despite the inclusion of the 80+ year age group, the correlation between both datasets remained relatively high at 94% (i.e *R²= 0.936*). The regression in the population stratified by age group after the exclusion of the 80+ year age group demonstrated an improvement to 99% correlation between both datasets.

**Table 1.** Summary of VCR and MA melanoma data for Victoria during the period 2004–2007 by gender. Number of unique patients for all age groups and excluding the 80+ year age group are listed.

	VCR Number of unique patients				MA Number of unique patients			
	All age groups		Excl. 80+ yr age group		All age groups		Excl. 80+ yr age group	
**Gender**	**#Patients**	**%**	**#Patients**	**%**	**#Patients**	**%**	**#Patients**	**%**
Male	8231	54.3	6950	53.9	7319	52.7	6691	53.5
Female	6919	45.7	5933	46.1	6567	47.3	5824	46.5
Total	15150	100.0	12883	100.0	13886	100.0	12515	100.0

**Figure 2.**
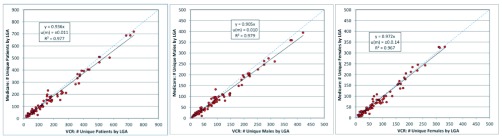
OLS regression of MA data against VCR melanoma data for the years 2004–2007 of all patients – stratified by LGA. ( **a**) all patients ( **b**) males ( **c**) females.

When stratified by LGA, comparisons of the VCR and MA datasets showed a close mapping when OLS regression was performed using both datasets in their entirety and when stratified by gender ( Figure 2). The gradient remained close to unity ( *m=*0.976 for the total dataset, *m*=0.905 for males and *m=*0.972 for females) with only a small standard of uncertainty (≈ u(m)±0.01). The *R*² values of 0.977, 0.979 and 0.967 respectively indicated a 97% correlation between the two datasets when all unique patients and cases by males and females were compared.

**Figure 3.**
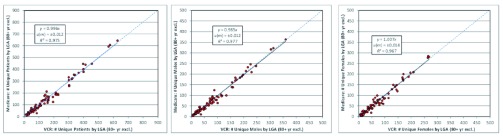
OLS regression of MA data against VCR melanoma data for the years 2004–2007, excluding patients >80 years old – stratified by LGA. ( **a**) all patients ( **b**) males ( **c**) females.

Statistical analysis was repeated on the entire dataset after excluding the 80+ year age group. Comparisons of the VCR and MA datasets after the exclusion of the 80+ year age group demonstrated a closer association between both datasets ( Figure 3). The gradient ( *m*=0.985) showed a closer degree of equivalence between the two datasets after this exclusion ( Figure 3). The *R²* were largely unaffected by the exclusion of the age group demonstrating that the correlation was high in both the male and female populations, regardless of the exclusion of the 80+ age group.

**Figure 4.**
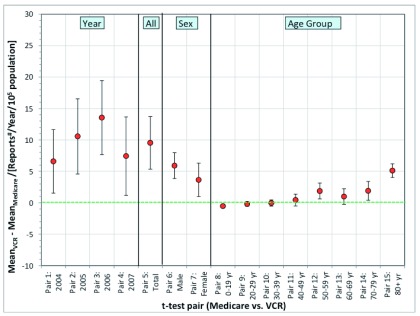
Plot diagram of VCR and MA mean difference incidence values for melanoma. Each data point represents the difference between the VCR and MA mean values for melanoma incidence. The uncertainty bars represent the 95% confidence interval.

The mean incidence data for the age groups 20–29, 30–39, 40–49 and 60–69 showed no statistical significance in the mean difference. The magnitude of mean difference for the remaining pairs was relatively low for patients in the 0–19, 50–59 and 70–79 year age group ( Figure 4).

## Discussion

Examining the ascertainment level MA MBS billing data for melanoma treatment is one step towards exploiting the vast body of epidemiological information collected on skin cancers by MA. In addition it has the potential for exploring other diseases which do not have mandatory reporting. The MA and VCR databases were examined looking at melanoma skin cancer. The findings of this study illustrate that the number of cases picked up by MA is comparable to the number of melanomas (malignant and *in situ*) registered with the VCR in patients above the age of 19 and below the age of 80 years old. Incidence values were also found to be similar in both datasets. This suggests that MA data may potentially be useful in examining melanoma trends. Furthermore, the findings represent the possibility of using this billing data to examine other types of skin cancer trends in Australia.

This study employed the use of VCR data, a large dataset for melanomas in Victoria. Mandatory reporting of melanomas is required in Australia and incidence and patterns are almost completely captured by population-based cancer registries ^26^. The large sample size of this dataset and variety of parameters (gender, age and LGA) strengthens the evaluation of the MA dataset. Overall, both the VCR and MA datasets showed a correlation when compared by year, gender and age. The 80+ year age group however was identified as a clear outlier. A potential reason for the discrepancy is the number of patients over the age of 80 who are billed by the Department of Veterans Affairs (DVA). Veterans, members of the Australian Defence Force and their spouses are eligible for a DVA health card which pays benefits for health care, pharmaceutical therapies and travel ^29^. Information regarding melanoma benefits paid by DVA was not included in the MA dataset.

This study is limited by the broad definitions of the MA item numbers. The melanoma item numbers used in this study covered the excision of the following: malignant melanomas, *in situ* melanomas (listed as Hutchinson’s freckle), appendageal carcinomas, malignant fibrous tumours of skin and Merkel cell carcinomas (MCC) ^27^. However the incidence rates of appendageal carcinomas, malignant fibrous tumours and MCC are low as these cancers are relatively rare ^30, 31^. Whilst no data exists on the exact figures of MCC Australia wide or within Victoria, estimates of 1 per 10 ^5^ men and 0.63 per 10 ^5^ women have been produced in a study in Western Australia ^30^. These low rates would not significantly affect the statistical results of this study and consequently the comparison between the MA item numbers and the VCR data was justified. However, the rates of MCC, fibrous tumours, malignant fibrous tumours and appendage carcinomas rise with age ^30^. In patients above the age of 85 the rates of MCC are much higher than the general population, occurring in 15.5 in 10 ^5^ people ^30^. The increase in these rarer skin cancers in the older age groups may also explain the discrepancy in the 80+ year age group. The analysis was conducted after excluding the 80+ year age group and this yielded a higher correlation. Furthermore, while melanomas are required to be histologically confirmed by MA, NMSC are not. GPs are required to obtain histological confirmation, in the case of an excision. Specialists, however, are permitted to bill MA without histological confirmation. This could potentially result in an over-estimate of lesions. Similarly, lesions that are treated with cryotherapy do not require histological verification, and this may also have resulted in an over-estimation by both GPs and specialists.

Since reporting of melanoma is mandatory by law, the data captured by cancer registries is therefore assumed to be sufficiently accurate to use for comparison purposes. Several epidemiological studies examining cancer databases compared capture rates to central cancer registries to determine ascertainment levels ^32, 33^. There are no figures published on the completeness of the VCR data. However, a study on the National Cancer Registry in Britain, which used a two-source capture-recapture method to estimate the number of cases in Britain and the fraction of registered patients, determined that the registry was 97–98% complete for melanoma registration; the assumption is that the capture rate in Australia would be similar ^32^.

MA has the potential to be used in order to examine NMSC trends. This potential could be explored further. NMSC are captured by registry within Tasmania and there is possibility of comparing MA data to NMSC registry data within Tasmania to further establish the ascertainment of MA ^1^. There is a paucity of data on incidence trends in pre-cancerous lesions such as actinic keratosis (AK) ^34^. Analysis of item numbers pertaining to the treatment of AK may potentially provide insight into these rates ^34^. Furthermore, longitudinal analysis of these lesions may help identify whether the introduction of newer agents including field treatments are more cost effective in treating AK.

## Conclusion

The current study explores the capture rate of MA for determining melanoma rate. The findings suggest that despite the inclusion of outlying patient groups, MA rebate data correlates closely with VCR data when assessing incidence calculation and other epidemiological measures for melanoma. NMSCs are not currently required to be reported to Australian cancer registries and thus use of such data may be used to capture NMSC cases, which represents a cost-effective method to establish trends. Longitudinal studies examining incidence trends and trends in residual and recurrent NMSC over several decades can examine the effectiveness of public health campaigns and consequential savings for future governments.

## Data availability

The data referenced by this article are under copyright with the following copyright statement: Copyright: © 2015 Perera E et al.

Raw datasets for MA and VCR are not available as they contain confidential information that cannot be deidentified. This data can be obtained by applying to the registry (MA or VCR) for access.

Some data about melanoma for the specific time period analysed is available from: http://medicarestatistics.humanservices.gov.au/statistics/mbs_item.jsp. This data includes the number of times an item number is billed, but does not provide unique identifiers (such as date of birth or location).
