# Supplementary material for: Validating the use of Medicare Australia billing data to examine trends in skin cancer
Source: F1000Res. 2015 Nov 24;4:1341. [Version 1] doi: 10.12688/f1000research.7161.1 (PMC4752029; doi:10.12688/f1000research.7161.1)
Supplement: Peer review history

# Peer review: Referee response for version 1

**DOI:** 10.5256/f1000research.7717.r12365

**Published:** 2016-02-11

**Authors:** Herbert Hönigsmann

**Affiliations:** 1 Department of Dermatology, Division of Special & Environmental Dermatology, Medical University of Vienna, Vienna, Austria

This report is well-written and appears sound. It is quite difficult to find appropriate statistic material on skin cancers in many countries. I see no problems.

I have read this submission. I believe that I have an appropriate level of expertise to confirm that it is of an acceptable scientific standard.

# Peer review: Referee response for version 1

**DOI:** 10.5256/f1000research.7717.r11331

**Published:** 2015-12-03

**Authors:** Mohamed Badawy Abdel-Naser

**Affiliations:** 1 Klinik für Dermatologie, Venerologie und Allergologie Städtisches Klinikum Dessau, Dessau, Germany

The study is well designed and the results are convincing.

The authors mentioned that the time period of data collection is from 2004 to 2008 which means 5 years (year 2008 is included). However in the table and figures the mentioned period was from 2004 to 2007 which means 4 year. Perhaps authors need to clarify this issue.

I have read this submission. I believe that I have an appropriate level of expertise to confirm that it is of an acceptable scientific standard.
